# Overcoming difficulties in duodenoscope insertion due to scope deflection by mounting a splinting tube on a therapeutic video duodenoscope

**DOI:** 10.1002/jhbp.12045

**Published:** 2024-07-19

**Authors:** Kyoko Asano, Yukitoshi Matsunami, Takao Itoi, Takayoshi Tsuchiya, Reina Tanaka, Ryosuke Tonozuka, Shuntaro Mukai, Hiroyuki Kojima, Eri Joyama, Atsushi Sofuni

**Affiliations:** ^1^ Department of Gastroenterology and Hepatology Tokyo Medical University Tokyo Japan; ^2^ Department of International Medical Care Tokyo Medical University Tokyo Japan

## Abstract

Asano and colleagues report their method of inserting a therapeutic video duodenoscope with the use of a splinting tube for challenging cases due to deformity of the pyloric antrum. With accompanying video, they demonstrate how this technique is promising for overcoming difficulties in duodenoscope insertion caused by scope deflection.

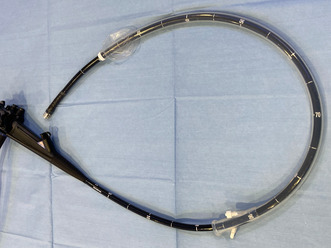

In pancreatobiliary endoscopy, inserting a duodenoscope may be challenging in some cases. Some overcoming methods have been reported, such as large balloon technique^1^, ^2^ and duodenoscope anchoring technique.^3^ In addition, very recently, scope insertion using a splinting tube method was reported.^4^ The use of a splinting tube (ST‐CB1; Olympus; inner diameter 13.8 mm) mounted on a conventional duodenoscope (JF‐260V; Olympus; maximum outer diameter 13.2 mm) made it easy to insert in a challenging case with perigastric adhesion. Based on this method, we report the successful insertion of a therapeutic video duodenoscope (TJF‐Q 290V; Olympus; maximum outer diameter 15.8 mm) with a splinting tube in a case of scope deflection due to deformity of the pyloric antrum (Video S1). A 67‐year‐old man underwent biliary stent replacement for malignant biliary obstruction due to unresectable pancreatic head cancer. The therapeutic video duodenoscope was initially inserted; however, the scope was deflected in the stomach while attempting to pass through the pyloric ring due to a deformity of the pyloric antrum caused by cancer. Therefore, we used a splinting tube mounted on the therapeutic duodenoscope to make the scope as firm as possible (Figure 1). By not attaching the disposable cover to the tip of the scope, which has the maximum diameter, we were able to smoothly insert the scope into the tube after filling it with water. Good firmness was maintained by advancing the scope with the tube, and the scope could be inserted into the duodenum without deflection in the stomach (Figure 2). Finally, the biliary stent was replaced without any adverse events (AEs). With careful attention to prevent AEs such as gastrointestinal perforation,^5^ this technique is promising for overcoming difficulties in duodenoscope insertion due to scope deflection.

**FIGURE 1:**
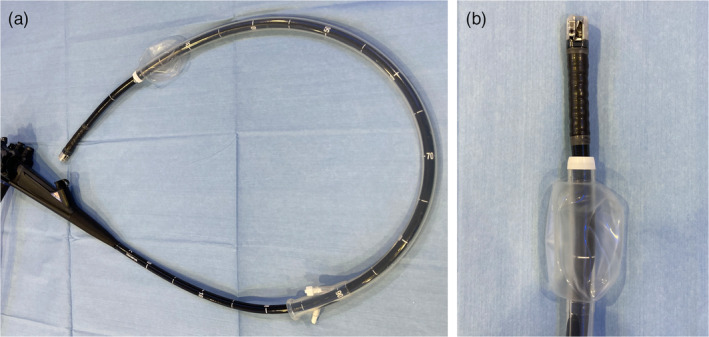
The single‐use splinting tube (ST‐CB1) mounted on the therapeutic video duodenoscope (TJF‐Q 290V). (a) Overview image. (b) Magnified image of the tip.

**FIGURE 2:**
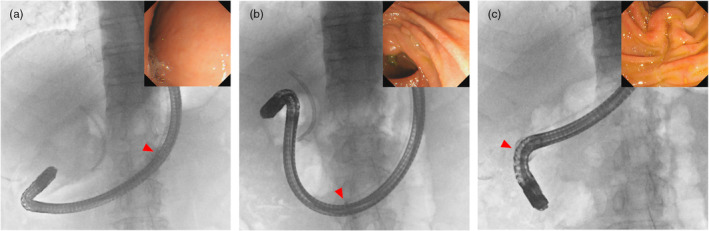
Fluoroscopic and endoscopic images of duodenoscope insertion using the splinting tube. Arrows indicate the head of the splinting tube. (a) The duodenoscope inserted into the pyloric antrum. (b) The duodenoscope passing the pyloric ring. (c) The duodenoscope at the major papilla with shortening position.

## CONFLICT OF INTEREST STATEMENT

Author T.I. received an honorarium for his lecture from Olympus. Other authors declare no conflicts of interest associated with this article.

## Supporting information


Video S1.
